# Design of Planar Differential Microphone Array Beampatterns with Controllable Mainlobe Beamwidth and Sidelobe Level

**DOI:** 10.3390/s23073733

**Published:** 2023-04-04

**Authors:** Xianghui Wang, Mei Li, Yingke Zhao, Jiao Wang, Xin Tan

**Affiliations:** 1 Center of Intelligent Acoustics and Immersive Communications, School of Electronic Information and Artificial Intelligence, Shaanxi University of Science and Technology, Xi’an 710021, China; 2 Shaanxi Joint Laboratory of Artificial Intelligence, Shaanxi University of Science and Technology, Xi’an 710021, China; 3 School of Electronic Information and Artificial Intelligence, Shaanxi University of Science and Technology, Xi’an 710021, China

**Keywords:** microphone array, fixed beamformer, differential beamformer, frequency-invariant beampattern, main lobe beamwidth, sidelobe level

## Abstract

The differential microphone array, or differential beamformer, has attracted much attention for its frequency-invariant beampattern, high directivity factor and compact size. In this work, the design of differential beamformers with small inter-element spacing planar microphone arrays is concerned. In order to exactly control the main lobe beamwidth and sidelobe level and obtain minimum main lobe beamwidth with a given sidelobe level, we design the desired beampattern by applying the Chebyshev polynomials at first, via exploiting the structure of the frequency-independent beampattern of a theoretical *N*th-order differential beamformer. Next, the so-called null constrained and least square beamformers, which can obtain approximately frequency-invariant beampattern at relatively low frequencies and can be steered to any direction without beampattern distortion, are proposed based on planar microphone arrays to approximate the designed desired beampatterns. Then, for dealing with the white noise amplification at low-frequency bands and beampattern divergence problems at high-frequency bands of the null constrained and least square beamformers, the so-called minimum norm and combined solutions are deduced, which can compromise among the white noise gain, directivity factor and beampattern distortion flexibly. Preliminary simulation results illustrate the properties and advantages of the proposed differential beamformers.

## 1. Introduction

Beamforming based on sensor/antenna array [1,2,3,4,5,6,7] has attracted much attention since the apparent advantage over the single sensor/antenna. The earliest beamforming technique is the delay-and-sum (DS) structure, which is investigated widely in narrowband applications [8,9,10,11], such as radar, sonar and antenna. However, the drawbacks of the varying beampattern with frequency and low directivity factor (DF) at low-frequency bands make the DS beamformer unsuitable for dealing with broadband signals, such as speech.

To overcome these drawbacks, many broadband beamforming methods have been developed, including the narrowband decomposition methods [12,13], the nested-array framework [14,15,16], the modal beamforming techniques [17,18,19,20], the super directive algorithms  [21,22,23] and the differential beamformers (or differential microphone arrays, DMAs)  [24,25,26,27,28,29]. Among those, the DMAs have been widely studied as they exhibit frequency-invariant beampatterns and high DF. Moreover, they are usually small and compact in size and consequently can be easily integrated into small devices, such as hearing aids, smartphones, smart wearable devices, etc. In this paper, we focus on the design of the DMAs.

The concept of the DMA evolutes from the directional ribbon microphone [30,31] responding to the sound pressure gradient field instead of the sound pressure field. The gradient direction of the sound field is parallel with the propagating direction of the sound, and the maximum directional derivative is in the gradient direction of the sound field. This is the underlying reason why we usually assume that the desired signal comes from the end-fire direction for a linear microphone array. Traditionally, the DMAs are designed in a cascaded subtraction way [32,33,34] by using several small inter-element spacing omnidirectional microphones. Through adjusting the time delay among different channels, many classical beampatterns can be obtained, including cardioid, dipole, supercardioid and hypercardioid. Based on the cascaded subtraction structure, much of the literature has been devoted to the design and study of the DMAs from different perspectives [34,35,36,37,38,39]. However, the shortcoming of the cascaded subtraction structure is inflexible in dealing with the white noise amplification problem, which is the inherent problem of differential beamformers and is very serious at low-frequency bands. It limits the deployment of the DMAs in practice.

In order to overcome the aforementioned drawback, a so-called null-constrained method was developed recently in [27,40] in the frequency domain, which enables one to design DMAs by only using the distortionless and null constraints. Then, a minimum norm solution is deduced in this framework by using more microphones to design a lower order DMA, and the problem of white noise amplification can be solved to a certain extent [27,40]. In [28], it is shown that the null-constrained method is the same as the cascaded subtraction way, and the minimum norm solution is also explained in a two-stage manner. Based on the null-constrained method, many interesting differential beamformers have been studied and developed [24,25,26,41,42,43,44].

Though much research about DMA has been done, further research attention is indispensable. For example, it is known that the main lobe beamwidth of the beampattern of the classical differential beamformer is usually large, especially the low-order ones. However, a narrower main lobe may be preferred in some applications for better source acquisition. In addition, in some particular scenarios, if we do not want to distort the background noise field very much, beampattern with the same level sidelobes is favored. So, how to obtain a beamformer with a narrower main lobe and how to exactly control the main lobe beamwidth and sidelobe level to obtain frequency-invariant beampatterns with expected characteristics are very important in DMA context, and to the best of our knowledge, these are still open issues.

In this work, for overcoming the above problems, we resort to the Chebyshev polynomials in the design of planar differential microphone array, which has been widely used in traditional array design area [45,46,47,48,49,50,51,52,53]. The main contributions of this manuscript are as follows. First, via investigating the frequency-independent beampattern of a theoretical *N*th-order differential beamformer, the desired beampattern is designed by exploiting the Chebyshev polynomials, whose main lobe beamwidth and sidelobe level can be exactly controlled, and the minimum main lobe beamwidth is guaranteed with a provided sidelobe level. Moreover, the relationship among the main lobe beamwidth, sidelobe level and polynomial order is deduced. Second, it has been proved theoretically in our framework that the first-order dipole beampattern has the narrowest beamwidth among the classical first-order differential beampatterns, i.e., dipole, cardioid, supercardioid and hypercardioid. Third, with the desired beampatterns at hands as targets, the so-called null constrained and least square beamformers for planar microphone array are proposed, which can obtain approximately frequency-invariant beampattern at relatively low frequencies and can be steered to any direction without beampattern distortion. Fourth, for overcoming the drawbacks of the null constrained and least square beamformers, i.e., severe white noise amplification problem at low frequencies and beampattern divergence from the desired one at high frequencies, the so-called minimum norm and combined solutions are deduced by exploiting the redundancy provided by using more microphones, which can flexibly compromise among the white noise gain (WNG), DF and beampattern divergence by adjusting parameters. Simulations are conducted and the preliminary results illustrate the properties and advantages of the proposed beamformers.

The remainder of this manuscript is as follows. The signal model, problem formulation and performance measures of beamforming are presented in Section 2. Section 3 introduces the approaches to the design of desired beampatterns. A set of differential beamformers are derived to approximate the desired beampatterns in Section 4. Simulations are conducted in Section 5, and conclusions are drawn in Section 6.

## 2. Signal Model, Problem Formulation and Performance Measures

**Figure 1:**
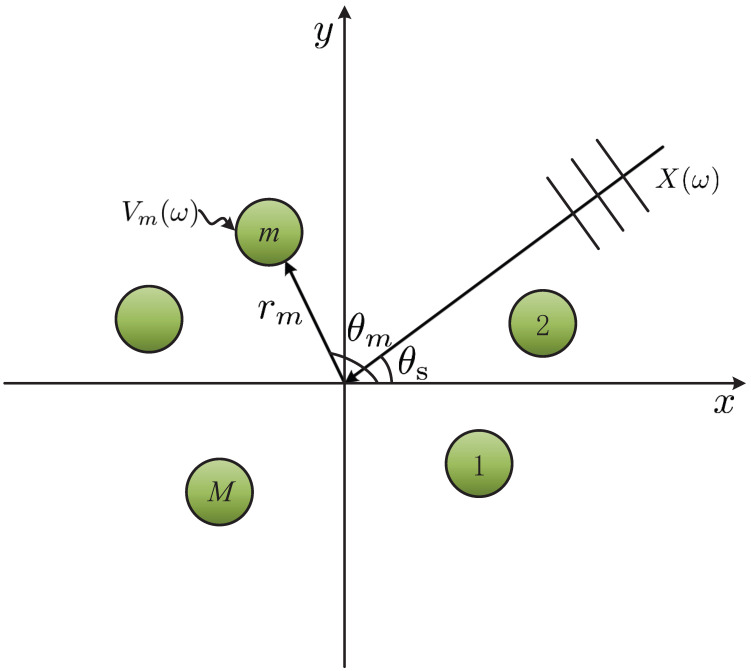
Illustration of a planar microphone array, where *M* is the microphone number, $\theta_{s}$ denotes the incidence angle of the desired source from farfield, $r_{m}$ denotes the distance between the *m*th microphone and the coordinate system origin, and $\theta_{m}$ is the angle of the *m*th microphone.

The farfield plane wave model is considered in this manuscript. The microphone array consists of *M* omnidirectional microphones which are placed in a horizontal plane as shown in Figure 1. ($r_{m}\cos\theta_{m}$, $r_{m}\sin\theta_{m}$) denotes the location of the *m*th sensor, whose radius and angle are denoted by $r_{m}$ and $\theta_{m}$, respectively. The desired source impinges on the microphone array from $\theta_{s}$ at the speed of sound c≈343 m/s. Obviously, the steering vector of the planar array is

(1) $$\begin{array}{cc} d(\omega ,\theta_{s}) & ={\left[\;e^{ȷ\omega r_{1}\cos\left(\theta_{s}-\theta_{1}\right)/c}\;\;\;e^{ȷ\omega r_{2}\cos\left(\theta_{s}-\theta_{2}\right)/c}\;\;\;\cdots \;\;\;e^{ȷ\omega r_{M}\cos\left(\theta_{s}-\theta_{M}\right)/c}\;\right]}^{T}, \end{array}$$

where the superscript ${}^{T}$ is the transpose operator, $ȷ=\sqrt{-1}$ is the imaginary unit, ω=2πf is the angular frequency, f>0 is the temporal frequency. Since the source is assumed to propagate from the angle $\theta_{s}$, the signals observed by the microphone array are given by

(2) $$\begin{array}{cc} y\left(\omega \right) & ={\left[\;Y_{1}\left(\omega \right)\;Y_{2}\left(\omega \right)\;\cdots \;Y_{M}\left(\omega \right)\;\right]}^{T}\\ \; & =x\left(\omega \right)+v\left(\omega \right)\\ \; & =d\left(\omega ,\theta_{s}\right)X\left(\omega \right)+v\left(\omega \right), \end{array}$$

where $Y_{m}\left(\omega \right)$ is the observed signal on *m*th microphone, $x\left(\omega \right)=d\left(\omega ,\theta_{s}\right)X\left(\omega \right)$, X(ω) is the zero-mean desired signal, and $v\left(\omega \right)$ is the zero-mean additive noise signal vector, which is defined similarly to $y\left(\omega \right)$. The desired signal and additive noise are assumed to be uncorrelated with each other. The beamformer output is then [1]

(3) $$\begin{array}{cc} Z\left(\omega \right) & =h^{H}\left(\omega \right)y\left(\omega \right)\\ \; & =h^{H}\left(\omega \right)d\left(\omega ,\theta_{s}\right)X\left(\omega \right)+h^{H}\left(\omega \right)v\left(\omega \right), \end{array}$$

where $Z\left(\omega \right)$ is the estimate of the desired signal, $X\left(\omega \right)$,

(4) $$\begin{array}{c} h\left(\omega \right)={\left[\;H_{1}\left(\omega \right)\;H_{2}\left(\omega \right)\;\cdots \;H_{M}\left(\omega \right)\;\right]}^{T} \end{array}$$

is a complex-valued linear filter applied to the observed signal vector, $y\left(\omega \right)$, and the superscript ${}^{H}$ denotes conjugate-transpose. In our context, we do not want the desired signal to be distorted by the beamformer, so the distortionless constraint is desired, i.e.,

(5) $$\begin{array}{c} h^{H}\left(\omega \right)d\left(\omega ,\theta_{s}\right)=1. \end{array}$$

The main interest of this work is beamforming with small inter-element spacing planar microphone arrays where the spacing between the neighboring microphones is much smaller than the minimum wavelength of the frequency band of interest. Consequently, the differentials of a different order of the acoustic pressure field can be approximated by finite differences in the microphones’ output. Usually, the objective of differential beamforming is to design beamformers, i.e., to find beamforming filters, $h\left(\omega \right)$, which response to the spatial derivative of acoustic pressure field of different orders, to estimate X(ω) as good as possible.

Usually, the three most important performance measures, i.e., beampattern, WNG and DF, are used to evaluate the fixed beamformer.

The beampattern or directivity pattern describes the sensitivity of the beamformer to a plane wave impinging on the array from the direction θ. Mathematically, it is given by

(6) $$\begin{array}{cc} B\left[h\left(\omega \right),\theta \right] & =d^{H}\left(\omega ,\theta \right)h\left(\omega \right)\\ \; & =\sum_{m=1}^{M}H_{m}\left(\omega \right)e^{-ȷ\omega r_{m}\cos\left(\theta -\theta_{m}\right)/c}. \end{array}$$

The null-to-null beamwidth of the beampattern is defined as the width between the two nulls which are closest to the main lobe from each side and is denoted by $B_{\mathrm{NN}}$.

With the first microphone being the reference (without loss of generality), the input signal-to-noise ratio (iSNR) is defined as

(7) $$\begin{array}{c} \mathrm{iSNR}\left(\omega \right)=\frac{\phi_{X}\left(\omega \right)}{\phi_{V_{1}}\left(\omega \right)}, \end{array}$$

where $\phi_{X}\left(\omega \right)=E\left[{\left|X\left(\omega \right)\right|}^{2}\right]$ and $\phi_{V_{1}}\left(\omega \right)=E\left[{\left|V_{1}\left(\omega \right)\right|}^{2}\right]$ are the variances of $X\left(\omega \right)$ and $V_{1}\left(\omega \right)$, respectively, and E[·] denotes the mathematical expectation. The output signal-to-noise ratio (oSNR), according to the beamforming model given in (3), is defined as

(8) $$\begin{array}{cc} \mathrm{oSNR}\left[h\left(\omega \right)\right] & =\phi_{X}\left(\omega \right)\frac{{\left|h^{H}\left(\omega \right)d\left(\omega ,\theta_{s}\right)\right|}^{2}}{h^{H}\left(\omega \right)\Phi_{v}\left(\omega \right)h\left(\omega \right)}\\ \; & =\frac{\phi_{X}\left(\omega \right)}{\phi_{V_{1}}\left(\omega \right)}\times \frac{{\left|h^{H}\left(\omega \right)d\left(\omega ,\theta_{s}\right)\right|}^{2}}{h^{H}\left(\omega \right)\Gamma_{v}\left(\omega \right)h\left(\omega \right)}, \end{array}$$

where $\Phi_{v}\left(\omega \right)=E\left[v\left(\omega \right)v^{H}\left(\omega \right)\right]$ and $\Gamma_{v}\left(\omega \right)=\frac{\Phi_{v}\left(\omega \right)}{\phi_{V_{1}}\left(\omega \right)}$ are the correlation and pseudo-coherence matrices of $v\left(\omega \right)$, respectively. The SNR gain derived from (7) and (8) is

(9) $$\begin{array}{cc} G\left[h\left(\omega \right)\right] & =\frac{\mathrm{oSNR}\left[h\left(\omega \right)\right]}{\mathrm{iSNR}\left(\omega \right)}\\ \; & =\frac{{\left|h^{H}\left(\omega \right)d\left(\omega ,\theta_{s}\right)\right|}^{2}}{h^{H}\left(\omega \right)\Gamma_{v}\left(\omega \right)h\left(\omega \right)}. \end{array}$$

Two particular types of noise are often considered for beamformer evaluation:

- Spatially and temporally white Gaussian noise with identical variances at all sensors, which models microphones’ self-noise, nonuniform responses among the microphones, imperfections of the microphone positions, etc. In this scenario, $\Gamma_{v}\left(\omega \right)=I_{M}$, where $I_{M}$ is the identity matrix of size M×M, and the resulting SNR gain is named as WNG, i.e.,
  (10) $$\begin{array}{c} W\left[h\left(\omega \right)\right]=\frac{{\left|h^{H}\left(\omega \right)d\left(\omega ,\theta_{s}\right)\right|}^{2}}{h^{H}\left(\omega \right)h\left(\omega \right)}. \end{array}$$
  So, the WNG evaluates the robust of the beamformer to some of the array imperfections. The maximum WNG that can be achieved by a beamforming filter is equal to *M* [40], i.e., $W_{\max}=M$. It can be obtained by the DS beamformer [40,54]:
  (11) $$\begin{array}{c} h_{\mathrm{DS}}\left(\omega \right)=\frac{d\left(\omega ,\theta_{s}\right)}{M}. \end{array}$$
  Note that $W\left[h\left(\omega \right)\right]<1$ indicates that there is white noise amplification.
- Spherical isotropic noise, which is characterized by
  (12) $$\begin{array}{cc} {\left[\Gamma_{d}\left(\omega \right)\right]}_{ij} & =\frac{\sin\left(\omega \delta_{ij}/c\right)}{\omega \delta_{ij}/c}=\mathrm{sinc}\left(\omega \delta_{ij}/c\right), \end{array}$$
  with ${\left[\Gamma_{d}\left(\omega \right)\right]}_{mm}=1,\;m=1,\;2,\;\ldots ,\;M$, where $\delta_{ij}$ is the spacing between the *i*th and *j*th microphones. In this case, the SNR gain is named DF:
  (13) $$\begin{array}{cc} D\left[h\left(\omega \right)\right]\; & =\frac{{\left|h^{H}\left(\omega \right)d\left(\omega ,\theta_{s}\right)\right|}^{2}}{h^{H}\left(\omega \right)\Gamma_{d}\left(\omega \right)h\left(\omega \right)}, \end{array}$$
  which quantifies how the beamformer performs in suppressing spatial directional noise, such as reverberation. The maximum DF that can be achieved by a beamformer with a given array is [40]
  (14) $$\begin{array}{c} D_{\max}\left(\omega \right)=d^{H}\left(\omega ,\theta_{s}\right)\Gamma_{d}^{-1}\left(\omega \right)d\left(\omega ,\theta_{s}\right), \end{array}$$
  which can be obtained by the so-called superdirective (hypercardioid) beamformer:
  (15) $$\begin{array}{c} h_{S}\left(\omega \right)=\frac{\Gamma_{d}^{-1}\left(\omega \right)d\left(\omega ,\theta_{s}\right)}{d^{H}\left(\omega ,\theta_{s}\right)\Gamma_{d}^{-1}\left(\omega \right)d\left(\omega ,\theta_{s}\right)}. \end{array}$$
  It can be shown that [55], when the uniform linear array is considered, we have
  (16) $$\begin{array}{c} \lim_{\delta \to 0}D_{\max}\left(\omega \right)=M^{2},\;\forall \omega , \end{array}$$
  which is referred as the supergain in the literature [56], and δ stands for the spacing between neighboring sensors.

## 3. Beampattern Design

It is well known that the frequency-independent beampattern of a theoretical *N*th-order DMA whose main lobe is at θ=0 can be written as [32,40]

(17) $$\begin{array}{c} B_{N}\left(\theta \right)=\sum_{n=0}^{N}a_{N,n}\cos^{n}\theta , \end{array}$$

where $a_{N,n},\;n=0,\;1,\;\ldots ,\;N$, are real coefficients that determine the beampattern shape. For normalization, we would like the beampattern to be 1 in the direction of the main lobe, i.e., $B_{N}\left(0\right)=1$. So, we have

(18) $$\begin{array}{c} \sum_{n=0}^{N}a_{N,n}=1. \end{array}$$

Different coefficients corresponding to different desired beampatterns of any order, such as classical dipole, cardioid, hypercardioid and supercardioid, can be found by optimizing different criteria [40,57], and one of the most important tasks of DMA design is to find desired beampattern and then approximate it as close as possible by beamforming filters.

While the aforementioned classical DMA beamformers have some optimum properties, there are some other issues. For example, the beamwidth of their beampattern is large usually, even for the hypercardioid beamformer which can obtain the maximum DF with a given microphone array. However, a narrower main lobe may be preferred in some applications for better source acquisition. In addition, in some particular scenarios, if we do not want to distort the background noise field very much, beampattern with the same level sidelobes is favored. So, in this manuscript, we try to exactly control the main lobe beamwidth and sidelobe level of the DMA beampattern.

From (17), we see that the beampattern, $B_{N}\left(\theta \right)$, is a polynomial of order *N* which is symmetric about θ=0. In order to exactly control the main lobe beamwidth and sidelobe level and obtain minimum main lobe beamwidth with a given sidelobe level, we resort to the Chebyshev polynomials, which have been widely used in traditional array signal processing area [46,54].

The Chebyshev polynomials, $T_{N}\left(x\right)=\cos\left(N\cos^{-1}x\right)$, are formed by using the Gram–Schmidt orthogonalization to the standard basis $1,\;x,\;x^{2},\;x^{3},\;\ldots$ in the interval x∈[−1,1] with respect to the weighting function $w\left(x\right)=1/\sqrt{1-x^{2}}$ [41], where $\cos^{-1}x$ is the inverse function of cosx. The first eight Chebyshev polynomials are [54]:

$$\begin{array}{cc} T_{0}\left(x\right) & =1,\\ T_{1}\left(x\right) & =x,\\ T_{2}\left(x\right) & =2x^{2}-1,\\ T_{3}\left(x\right) & =4x^{3}-3x,\\ T_{4}\left(x\right) & =8x^{4}-8x^{2}+1,\\ T_{5}\left(x\right) & =16x^{5}-20x^{3}+5x,\\ T_{6}\left(x\right) & =32x^{6}-48x^{4}+18x^{2}-1,\\ T_{7}\left(x\right) & =64x^{7}-112x^{5}+56x^{3}-7x, \end{array}$$

and for N≥2, we have

(19) $$\begin{array}{c} T_{N}\left(x\right)=2xT_{N-1}\left(x\right)-T_{N-2}\left(x\right). \end{array}$$

The Chebyshev polynomials are a set of functions that are orthogonal in [−1,1] with respect to the weighting function w(x) [41], i.e.,

(20) $$\begin{array}{c} \int_{-1}^{1}\frac{1}{\sqrt{1-x^{2}}}T_{n}\left(x\right)T_{m}\left(x\right)dx=c_{n}\delta \left(n-m\right), \end{array}$$

where δ(·) is the Dirac delta function, and $c_{n}$ is

(21) $$\begin{array}{c} c_{n}=\left\{\begin{array}{cc} \pi , & n=0\\ \pi /2, & n\neq 0 \end{array}\right.. \end{array}$$

The definition of the *N*th degree Chebyshev polynomial on the whole range of *x* is

(22) $$\begin{array}{c} T_{N}\left(x\right)=\left\{\begin{array}{cc} \cos\left(N\cos^{-1}x\right), & \left|x\right|\leq 1\\ \cosh\left(N\cosh^{-1}x\right), & x>1\\ {\left(-1\right)}^{N}\cosh\left(N\cosh^{-1}x\right), & x<-1 \end{array}\right., \end{array}$$

where $\cosh^{-1}x$ is the inverse function of coshx.

$T_{N}\left(x\right)$ has *N* real roots in x∈[−1,1]. By taking x=cosθ, we know that the roots are at cos(Nθ)=0, i.e.,

(23) $$\begin{array}{c} N\theta =\left(2k-1\right)\frac{\pi }{2},\;1\leq k\leq N. \end{array}$$

From (22), we know that the Chebyshev polynomials have equal ripple characteristics in the interval [−1,1] (the magnitude of every maxima and minima is 1, i.e., $|T_{N}\left(x\right)|\leq 1$), and all of the polynomials pass through the points (1,1) and $\left(-1,\;{\left(-1\right)}^{N}\right)$. When |x|>1, we have $|T_{N}\left(x\right)|>1$.

We assume the magnitude of the main lobe corresponds to the value of $T_{N}\left(x_{0}\right)$ where $x_{0}>1$. The ratio of the main lobe level to the sidelobe level is defined as *R*, i.e.,

(24) $$\begin{array}{c} R=\frac{L_{m}}{L_{s}}, \end{array}$$

where $L_{m}$ and $L_{s}$ denote the main lobe level and sidelobe level, respectively. So, the point $\left(x_{0},\;R\right)$ on the $T_{N}\left(x\right)$ curve is the position of the mainlobe maximum. According to (22), we have

(25) $$\begin{array}{c} x_{0}=\cosh\left(\frac{1}{N}\cosh^{-1}R\right). \end{array}$$

If we simply use x=cosθ, then |x|≤1. However, in order to design the desired beampattern, we should utilize the entire range $\left[-1,\;x_{0}\right]$ of the Chebyshev polynomial [46,54]. So, we assume

(26) $$\begin{array}{c} x=c_{1}\cos\theta +c_{2}, \end{array}$$

where $c_{1}$ and $c_{2}$ are constant values. The range $\left[-1,\;x_{0}\right]$ in *x*-space corresponds to the range [0,π] in θ-space. So, in order to control the whole range of the beampattern, i.e., θ∈[0,π], the mapping relationship presented in Table 1 should be satisfied [46].

To determine $c_{1}$ and $c_{2}$, we solve the following two equations:

$$\begin{array}{cc} c_{1}+c_{2} & =x_{0},\\ -c_{1}+c_{2} & =-1, \end{array}$$

and have

(27) $$\begin{array}{c} c_{1}=\frac{x_{0}+1}{2},\;c_{2}=\frac{x_{0}-1}{2}. \end{array}$$

Substituting (27) into (26), we have

(28) $$\begin{array}{c} x=\frac{x_{0}+1}{2}\cos\theta +\frac{x_{0}-1}{2}. \end{array}$$

So, the desired beampattern is

(29) $$\begin{array}{c} B_{N,d}\left(\theta \right)=\frac{1}{R}T_{N}\left(\frac{x_{0}+1}{2}\cos\theta +\frac{x_{0}-1}{2}\right), \end{array}$$

where the factor 1/R normalizes the beampattern making $B_{N}\left(0\right)=1$. Obviously, we can steer the desired beampattern to $\theta_{s}$ as

(30) $$\begin{array}{c} B_{N,d}\left(\theta -\theta_{s}\right)=\frac{1}{R}T_{N}\left[\frac{x_{0}+1}{2}\cos\left(\theta -\theta_{s}\right)+\frac{x_{0}-1}{2}\right]. \end{array}$$

For conciseness, in this section, we only discuss the case of $\theta_{s}=0$, i.e., the desired beampattern in (29). A simple beampattern steering method will be given in the next section.

From (23), we know that the roots of $T_{N}\left(\cos\theta \right)$ are

(31) $$\begin{array}{c} \theta_{k}=\frac{(2k-1)\pi }{2N},\;1\leq k\leq N. \end{array}$$

So, in *x*-space the roots are at

(32) $$\begin{array}{c} x=\frac{x_{0}+1}{2}\cos\theta +\frac{x_{0}-1}{2}=\cos\theta_{k}. \end{array}$$

Then, we know the roots in the scaled space occur at

(33) $$\begin{array}{cc} \; & \theta_{k0}=\cos^{-1}\left[\frac{2}{x_{0}+1}\left(\cos\theta_{k}-\frac{x_{0}-1}{2}\right)\right],\;k=1,\;2,\;\ldots ,\;N. \end{array}$$

From (33), we know the main lobe beamwidth is

(34) $$\begin{array}{c} B_{\mathrm{NN}}=2\theta_{10}=2\cos^{-1}\left[\frac{2}{x_{0}+1}\left(\cos\frac{\pi }{2N}-\frac{x_{0}-1}{2}\right)\right]. \end{array}$$

By simple manipulation, we obtain

(35) $$\begin{array}{cc} B_{\mathrm{NN}}\; & =2\cos^{-1}\left[\frac{2}{x_{0}+1}\left(\cos\frac{\pi }{2N}+1\right)-1\right]\\ \; & =2\cos^{-1}\left[\frac{2}{\cosh\left(\frac{1}{N}\cosh^{-1}R\right)+1}\left(\cos\frac{\pi }{2N}+1\right)-1\right]. \end{array}$$

Since $x_{0}>1$ and N≥1, we have

(36) $$\begin{array}{c} -1<\frac{2}{x_{0}+1}\left(\cos\frac{\pi }{2N}+1\right)-1<1. \end{array}$$

So, we can observe that the beamwidth, $B_{\mathrm{NN}}$, increases with *R* (which increases with the increase of $x_{0}$) and decreases with *N*. Consequently, we can make a compromise between the main lobe beamwidth and sidelobe level with a provided *N* flexibly. When *N* is given, the main lobe beamwidth can be adjusted by tuning *R*, and the minimum beamwidth is obtained when R→1 (i.e., $x_{0}\to 1$), which means the magnitudes of the main lobe and sidelobe are the same, and we have

(37) $$\begin{array}{c} \lim_{R\to 1}B_{\mathrm{NN}}=\frac{\pi }{N}. \end{array}$$

So, the 1st order dipole beampattern, which has the same magnitudes of the main lobe and sidelobe and a null at π/2, has the minimum main lobe beamwidth, i.e., $B_{\mathrm{NN}}=\pi$, among the different 1st order DMA beampatterns.

From (35), we can also get

(38) $$\begin{array}{c} R=\cosh\left\{N\cosh^{-1}\left[\frac{2\left(\cos\frac{\pi }{2N}+1\right)}{\cos\frac{B_{\mathrm{NN}}}{2}+1}-1\right]\right\}, \end{array}$$

i.e., when *N* and $B_{\mathrm{NN}}$ are given, we can determine *R* using (38). However, it should be noted that the $B_{\mathrm{NN}}$ will never be narrower than π/N. The method of designing the desired beampattern is described in Algorithm 1.
**Algorithm 1:** Steps for designing the desired beampattern.1: Select the order, *N*, of the desired beampattern, $B_{N}\left(\theta \right)$, and choose the Chebyshev polynomial, $T_{N}\left(x\right)$, of the same order;2: Set the ratio of the main lobe level to the sidelobe level, *R*, and determine $x_{0}$ according to (25), or set the main lobe beamwidth $B_{\mathrm{NN}}$, and determine the parameter *R* according to (38) and then find $x_{0}$;3: Change the scale by using the mapping function (28);4: Obtain the desired beampattern, $B_{N,d}\left(\theta \right)$, according to (29) (the methods of approximating the desired beampattern are described in Section 4).

It is observed that, from (35), we can calculate the order of the Chebyshev polynomial, $N_{F}$, by using numerical methods when $B_{\mathrm{NN}}$ and *R* are given, where the subscript `F’ denotes fractional. Though the polynomial order *N* should be an integer, $N_{F}$ gives us a good reference.

As seen, (29) can be rewritten as

(39) $$\begin{array}{c} B_{N,d}\left(\theta \right)=\frac{1}{R}T_{N}\left[\frac{x_{0}+1}{4}\left(e^{ȷ\theta }+e^{-ȷ\theta }\right)+\frac{x_{0}-1}{2}\right], \end{array}$$

and the component $e^{-ȷ\omega r_{m}\cos\left(\theta -\theta_{m}\right)/c}$ in (6) can be expressed as a function of $e^{-ȷn\theta }$ using the Jacobi-Anger expansion [58,59]. Consequently, we can deduce the beamformer coefficients by solving the following equation:

(40) $$\begin{array}{c} B\left[h\left(\omega \right),\theta \right]=B_{N,d}\left(\theta \right). \end{array}$$

However, it is difficult when *N* is large. So, for simplicity, we proposed the so-called null-constrained and least square beamformers in the next section.

## 4. DMA Design

With the desired beampattern, $B_{N,d}\left(\theta \right)$, at hand as target, our task is to approximate it as close as possible. In this work, we first introduce two kinds of methods, i.e., null-constrained and least square methods. Then, we derive the minimum norm and combined solutions in order to improve the white noise amplification problem at low-frequency bands and prevent the array beampattern from significantly deviating from the desired one at high-frequency bands.

From (17), we know the desired beampattern is symmetric about the 0↔π axis. However, to design the beampattern of the planar array, we need to consider the whole range of θ, i.e., θ∈[0,2π]. So, 2N zeros are taken in to account in our context, i.e., $\theta_{k0},\;k=1,\;2,\;\ldots ,\;2N$. The first *N* zeros are $\theta_{k0},\;k=1,\;2,\;\ldots ,\;N$, and the last *N* zeros are $2\pi -\theta_{k0},\;k=1,\;2,\;\ldots ,\;N$. It should be noted that, since $T_{N}\left(x\right)$ pass through the point $\left(-1,\;{\left(-1\right)}^{N}\right)$, we will never have a null at θ=π, with a given limited *R*. So, there is not higher (than 1) order null.

### 4.1. Null Constrained Method

As known, the position of nulls is very important in DMA design, which determines the beampattern of DMA uniquely [27,40]. In this part, we assume $M=M_{N}=2N+1$, where $M_{N}$ is the minimum microphone number that is required to form a *N*th order planar DMA beampattern given the array geometry. By combining the distortionless constraint (which is at θ=0) and the null constraints, we have the following linear system:

(41) $$\begin{array}{c} D\left(\omega ,\theta_{0}\right)h\left(\omega \right)=i_{M_{N}}, \end{array}$$

where

(42) $$\begin{array}{c} D\left(\omega ,\theta_{0}\right)=\left[\begin{array}{c} d^{H}\left(\omega ,0\right)\\ d^{H}\left(\omega ,\theta_{10}\right)\\ d^{H}\left(\omega ,\theta_{20}\right)\\ \vdots \\ d^{H}\left(\omega ,\theta_{N0}\right)\\ \vdots \\ d^{H}\left(\omega ,\theta_{2N0}\right) \end{array}\right] \end{array}$$

is the constraint matrix of size $M_{N}\times M_{N}$, which is assumed to be a full rank matrix, $\theta_{0}=\left[\;\theta_{10},\;\theta_{20},\;\cdots ,\;\theta_{N0},\;\cdots ,\;\theta_{2N0}\;\right]$ and $i_{M_{N}}$ is the first column of the identity matrix, $I_{M_{N}}$, of size $M_{N}\times M_{N}$. So, we can obtain the beamformer as

(43) $$\begin{array}{c} h_{\mathrm{NC}}\left(\omega \right)=D^{-1}\left(\omega ,\theta_{0}\right)i_{M_{N}}. \end{array}$$

If we want to steer the main lobe to directions other than θ=0, the nulls can be set at $\theta_{0}=\left[\;\theta_{s}+\theta_{10},\;\theta_{s}+\theta_{20},\;\cdots ,\;\theta_{s}+\theta_{N0},\;\cdots ,\;\theta_{s}+\theta_{2N0}\;\right]$, and now the distortionless constraint is $d^{H}\left(\omega ,\theta_{s}\right)h\left(\omega \right)=1$, where $\theta_{s}$ is the steered direction.

### 4.2. Least Square Method

In this part, again, we assume $M=M_{N}$. Let us define the error of the DMA beampattern first:

(44) $$\begin{array}{cc} E\left[h(\omega ),\;\theta \right] & =B_{N,d}\left(\theta \right)-B\left[h\left(\omega \right),\theta \right]\\ \; & =B_{N,d}\left(\theta \right)-d^{H}\left(\omega ,\theta \right)h\left(\omega \right). \end{array}$$

Then, the least square approximation criterion [41] can be written as

(45) $$\begin{array}{cc} J_{\mathrm{LS}}\left[h(\omega )\right] & =\int_{0}^{2\pi }{\left|E\left[h(\omega ),\;\theta \right]\right|}^{2}d\theta \\ \; & =h^{H}\left(\omega \right)P\left(\omega \right)h\left(\omega \right)-h^{H}\left(\omega \right)q\left(\omega \right)-q^{H}\left(\omega \right)h\left(\omega \right)+\int_{0}^{2\pi }{\left|B_{N,d}\left(\theta \right)\right|}^{2}d\theta , \end{array}$$

where

$$\begin{array}{cc} P(\omega ) & =\int_{0}^{2\pi }d\left(\omega ,\theta \right)d^{H}\left(\omega ,\theta \right)d\theta ,\\ q(\omega ) & =\int_{0}^{2\pi }B_{N,d}\left(\theta \right)d\left(\omega ,\theta \right)d\theta \end{array}$$

are matrices of size $M_{N}\times M_{N}$ and $M_{N}\times 1$, respectively. Of course, we can use a weighting function to either emphasize or deemphasize some specific angles. However, for simplicity, we set the weighting function to 1 in this work. By combining $J_{\mathrm{LS}}\left[h(\omega )\right]$ and the distortionless constraint, we obtain the cost function, i.e.,

(46) $$\begin{array}{cc} \; & \min_{h\left(\omega \right)}J_{\mathrm{LS}}\left[h(\omega )\right]\;\;\mathrm{subject}\;\mathrm{to}\;\;\;d^{H}\left(\omega ,0\right)h\left(\omega \right)=1. \end{array}$$

Solving (46) by using the Lagrange multiplier method, the beamformer is derived as

(47) $$\begin{array}{c} h_{\mathrm{LS}}\left(\omega \right)=\frac{P^{-1}\left(\omega \right)q\left(\omega \right)}{q^{H}\left(\omega \right)P^{-1}\left(\omega \right)d\left(\omega ,0\right)}. \end{array}$$

In this case, if we want to steer the main lobe to direction $\theta_{s}\neq 0$, we can set the desired beampattern as in (30).

### 4.3. Minimum Norm Solution

In the traditional and the above DMA design routines, it is assumed that $M=M_{N}$. As known, one of the inherent shortcomings of the differential beamformer is the white noise amplification problem at low-frequency bands. Recently, in order to improve this thorny problem, the minimum norm solution is proposed in [40,57] which takes the advantage that we can exploit the redundancy by using more microphones, i.e., $M>M_{N}$. In this context, the linear system can be written as

(48) $$\begin{array}{c} \bar{D}\left(\omega ,\theta_{0}\right)h\left(\omega \right)=i_{M_{N}}, \end{array}$$

where

(49) $$\begin{array}{c} \bar{D}\left(\omega ,\theta_{0}\right)=\left[\begin{array}{c} {\bar{d}}^{H}\left(\omega ,0\right)\\ {\bar{d}}^{H}\left(\omega ,\theta_{10}\right)\\ {\bar{d}}^{H}\left(\omega ,\theta_{20}\right)\\ \vdots \\ {\bar{d}}^{H}\left(\omega ,\theta_{N0}\right)\\ \vdots \\ {\bar{d}}^{H}\left(\omega ,\theta_{2N0}\right) \end{array}\right] \end{array}$$

is the constraint matrix of size $M_{N}\times M$, which is assumed to be a row full rank matrix, and $\bar{d}\left(\omega ,\theta \right)$ is the steering vector of length $M>M_{N}$. Then, the minimum norm solution can be derived by solving the following cost function:

(50) $$\begin{array}{cc} \; & \min_{h\left(\omega \right)}h^{H}\left(\omega \right)h\left(\omega \right)\;\;\;\mathrm{subject}\;\mathrm{to}\;\;\;\bar{D}\left(\omega ,\theta_{0}\right)h\left(\omega \right)=i_{M_{N}}, \end{array}$$

and the beamforming filter is

(51) $$\begin{array}{c} h_{\mathrm{MN}}\left(\omega \right)={\bar{D}}^{H}\left(\omega ,\theta_{0}\right){\left[\bar{D}\left(\omega ,\theta_{0}\right){\bar{D}}^{H}\left(\omega ,\theta_{0}\right)\right]}^{-1}i_{M_{N}}. \end{array}$$

### 4.4. Combined Solution

Usually, at relatively high-frequency bands (compared to the spacing between the neighboring sensors), the array beampattern significantly deviates from the desired one since the differentials of a different order of the acoustic pressure field can no longer be approximated precisely by finite difference of the microphones’ output when the microphone array geometry is given. So, it is necessary to control the distortion of the beampattern and the white noise amplification problem simultaneously. Consequently, we apply the following cost function [41]:

(52) $$\begin{array}{cc} \; & \min_{h\left(\omega \right)}\mu h^{H}\left(\omega \right)h\left(\omega \right)+\left(1-\mu \right)J_{\mathrm{LS}}\left[h(\omega )\right]\;\;\;\mathrm{subject}\;\mathrm{to}\;\;\;\bar{D}\left(\omega ,\theta_{0}\right)h\left(\omega \right)=i_{M_{N}}, \end{array}$$

and the solution which is named a combined solution is

(53) $$\begin{array}{cc} \; & h_{C}\left(\omega \right)={\bar{P}}_{\mu }^{-1}\left(\omega \right)\left\{2\left(1-\mu \right)\bar{q}\left(\omega \right)+{\bar{D}}^{H}\left(\omega ,\theta_{0}\right){\bar{Q}}_{\mu }^{-1}\left(\omega \right)\left[i_{M_{N}}-2\left(1-\mu \right)\bar{D}\left(\omega ,\theta_{0}\right){\bar{P}}_{\mu }^{-1}\left(\omega \right)\bar{q}\left(\omega \right)\right]\right\}, \end{array}$$

where

$$\begin{array}{cc} {\bar{P}}_{\mu }\left(\omega \right) & =2\mu I_{M}+2\left(1-\mu \right)\bar{P}\left(\omega \right),\\ {\bar{Q}}_{\mu }\left(\omega \right) & =\bar{D}\left(\omega ,\theta_{0}\right){\bar{P}}_{\mu }^{-1}\left(\omega \right){\bar{D}}^{H}\left(\omega ,\theta_{0}\right),\\ \bar{P}\left(\omega \right) & =\int_{0}^{2\pi }\bar{d}\left(\omega ,\theta \right){\bar{d}}^{H}\left(\omega ,\theta \right)d\theta ,\\ \bar{q}\left(\omega \right) & =\int_{0}^{2\pi }B_{N,d}\left(\theta \right)\bar{d}\left(\omega ,\theta \right)d\theta \end{array}$$

are of size M×M, $M_{N}\times M_{N}$, M×M and M×1, respectively, and 0≤μ≤1. When μ=1, we get the minimum norm solution, $h_{\mathrm{MN}}\left(\omega \right)$.

For more degree of freedom to deal with the white noise amplification problem at low frequencies, we simplify (52) as [41]

(54) $$\begin{array}{c} \min_{h\left(\omega \right)}\mu h^{H}\left(\omega \right)h\left(\omega \right)+\left(1-\mu \right)J_{\mathrm{LS}}\left[h(\omega )\right]\;\;\;\mathrm{subject}\;\mathrm{to}\;\;\;{\bar{d}}^{H}\left(\omega ,0\right)h\left(\omega \right)=1, \end{array}$$

whose solution is

(55) $$\begin{array}{cc} \; & h_{\mathrm{CF}}\left(\omega \right)={\bar{P}}_{\mu }^{-1}\left(\omega \right)\left[2\left(1-\mu \right)\bar{q}\left(\omega \right)+\frac{1-2\left(1-\mu \right){\bar{d}}^{H}\left(\omega \right){\bar{P}}_{\mu }^{-1}\left(\omega \right)\bar{q}\left(\omega \right)}{{\bar{d}}^{H}\left(\omega \right){\bar{P}}_{\mu }^{-1}\left(\omega \right)\bar{d}\left(\omega \right)}\bar{d}\left(\omega \right)\right]. \end{array}$$

When μ=0, we get the least square beamformer, $h_{\mathrm{LS}}\left(\omega \right)$, with redundant microphone, and when μ=1, we get the delay-and-sum beamformer, $h_{\mathrm{DS}}\left(\omega \right)$.

### 4.5. Particular Case: Linear Microphone Array

The methods proposed above are applicable to linear microphone arrays. However, for a linear array, we only need to consider the range θ∈[0,π]. In this case, the microphone number *M* is require to satisfy $M\geq M_{N,L}=N+1$, $\theta_{0}=\left[\;\theta_{10},\;\theta_{20},\;\cdots ,\;\theta_{N0}\;\right]$, and $D(\omega ,\theta_{0})$ of size $M_{N,L}\times M_{N,L}$ and $\bar{D}\left(\omega ,\theta_{0}\right)$ of size $M_{N,L}\times M$ are, respectively,

(56) $$\begin{array}{c} D\left(\omega ,\theta_{0}\right)=\left[\begin{array}{c} d^{H}\left(\omega ,0\right)\\ d^{H}\left(\omega ,\theta_{10}\right)\\ d^{H}\left(\omega ,\theta_{20}\right)\\ \vdots \\ d^{H}\left(\omega ,\theta_{N0}\right) \end{array}\right] \end{array}$$

and

(57) $$\begin{array}{c} \bar{D}\left(\omega ,\theta_{0}\right)=\left[\begin{array}{c} {\bar{d}}^{H}\left(\omega ,0\right)\\ {\bar{d}}^{H}\left(\omega ,\theta_{10}\right)\\ {\bar{d}}^{H}\left(\omega ,\theta_{20}\right)\\ \vdots \\ {\bar{d}}^{H}\left(\omega ,\theta_{N0}\right) \end{array}\right]. \end{array}$$

It should be noted that the steering ability of the linear DMA is limited [60,61,62].

## 5. Simulations

In this section, we evaluate the performance of the methods proposed above.

### 5.1. Desired Beampattern

**Figure 2:**
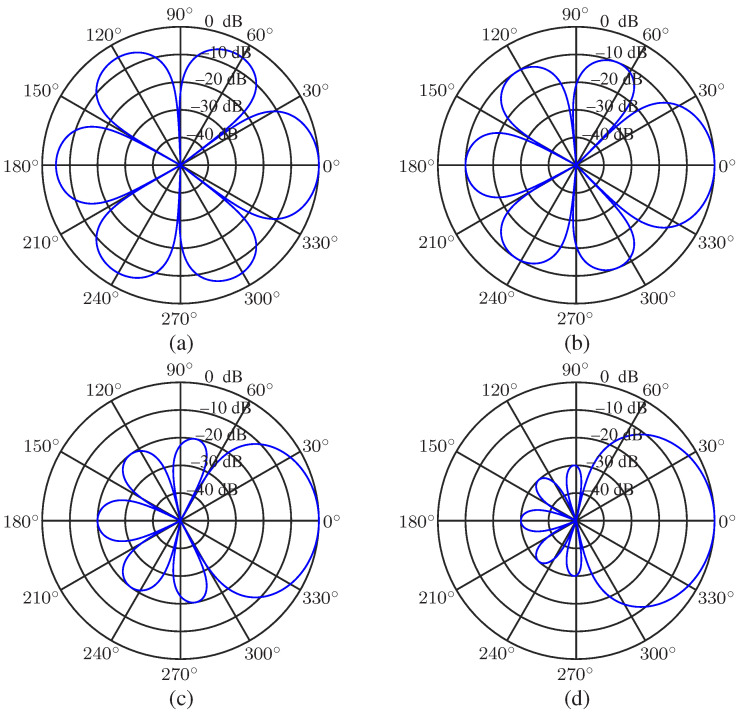
Desired beampatterns with different ratio, *R*, of mainlobe level to sidelobe level: (**a**) R=5, (**b**) R=10, (**c**) R=20, and (**d**) R=30. Conditions: N=3, and $\theta_{s}=0$.

**Figure 3:**
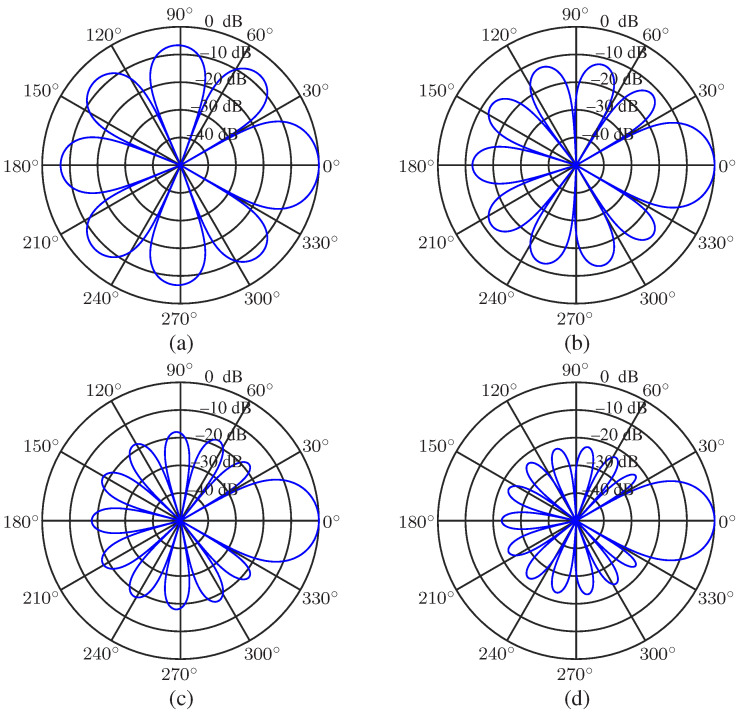
Desired beampatterns with a given mainlobe beamwidth $B_{\mathrm{NN}}$ and different polynomial order *N*: (**a**) N=4, (**b**) N=5, (**c**) N=6, and (**d**) N=7. Conditions: $B_{\mathrm{NN}}=\pi /3$ and $\theta_{s}=0$.

We first study the desired beampattern with a different main lobe level to sidelobe level ratio *R*. The order of the Chebyshev polynomial is set to be N=3, and R=5,10,20 and 30 (in dB, the same below) are considered. The results are presented in Figure 2. As seen, the sidelobe level is controlled precisely, and the main lobe beamwidth increases with the increase of *R*. When *R* is small, the main lobe beamwidth is narrower than that of the superdirective beamformer (whose beampattern can be found in [21,27]) Next, we investigate the beampatterns with a given main lobe beamwidth $B_{\mathrm{NN}}=\pi /3$ and different polynomial order *N* (N=4,5,6,7). The beampatterns are plotted in Figure 3. It is observed that we can exactly control the main lobe beamwidth if the polynomial order *N* is adequate.

Then, we evaluate the performance of the beamformers proposed in Section 4.

### 5.2. Performance of the Null Constrained and Least Square Beamformers

**Figure 4:**
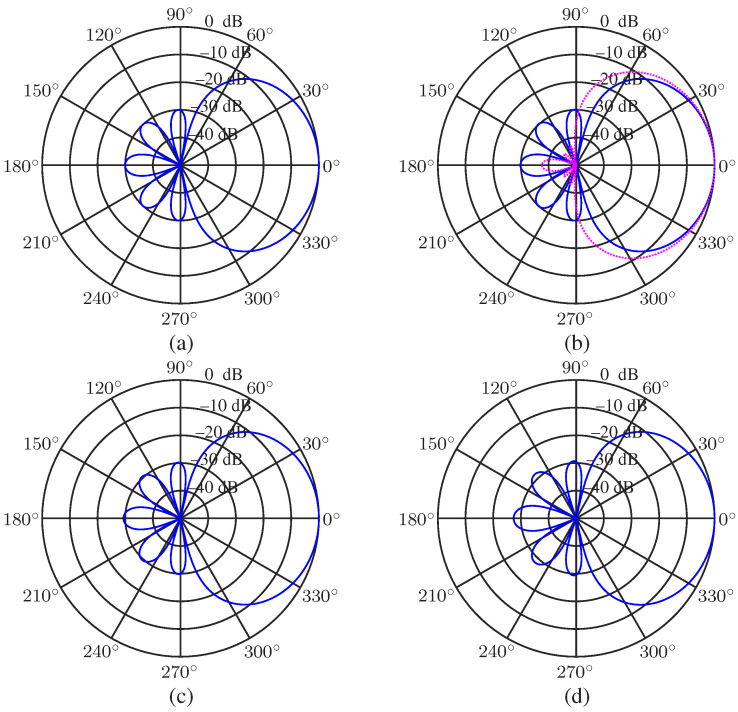
Beampatterns of the null constrained beamformer, $h_{\mathrm{NC}}\left(\omega \right)$, with different frequencies: (**a**) f=500 Hz, (**b**) f=1000 Hz, (**c**) f=2000 Hz, and (**d**) f=4000 Hz. Conditions: N=3, M=7, R=30, r=0.02 m, and $\theta_{s}=0$.

**Figure 5:**
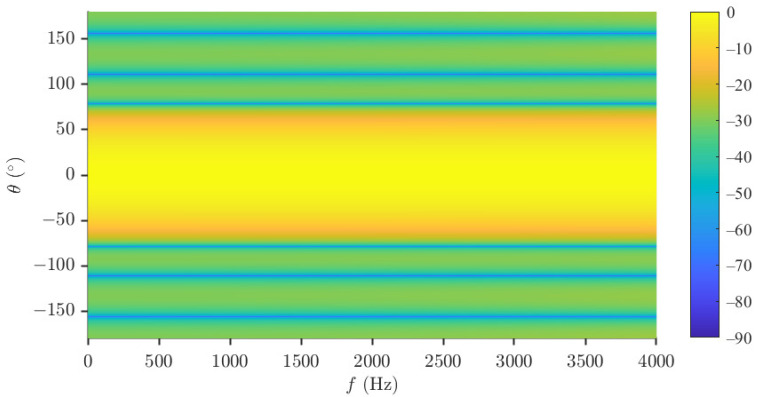
Beampattern of the null constrained beamformer, $h_{\mathrm{NC}}\left(\omega \right)$, versus frequency. Conditions: N=3, M=7, R=30, r=0.02 m, and $\theta_{s}=0$.

The uniform circular array (UCA) is considered in the performance evaluation parts. The null-constrained beamformer is studied first. Again, the order of the Chebyshev polynomial is set to be N=3, and R=30 (dB) is taken, i.e., the desired beampattern that we want to approximate is Figure 2d. So, M=7 microphones are needed. The radius of the UCA is set to be r=0.02 m. The beampatterns of the null-constrained beamformer with different frequencies (f=500,1000,2000 and 4000 Hz) are shown in Figure 4, including the beampattern of the maximum front-to-back ratio [63] (purple dot line) beamformer at f=1000 Hz. We can see from Figure 4 that, at relatively low frequencies, the beampattern of the null-constrained beamformer is very close to the desired one. However, at high frequencies, the array beampattern diverges from the desired beampattern gradually, and the divergence increases with the increase of frequency. The reason has been explained in Section 4.4. In addition, as we can see from Figure 4b, comparing with the beampattern of the maximum front-to-back ratio beamformer, the sidelobe level of $h_{\mathrm{NC}}\left(\omega \right)$ is exactly controlled, and the level of different sidelobe is the same, which is useful if we do not want to distort the background noise field too much. Figure 5 plots the 3-D beampattern of the null constrained beamformer versus frequency with $\theta_{s}=0$. We can see that the beampattern is approximate frequency-invariant at low frequencies, and the sidelobe level gradually increases with the increase of frequency at high frequencies.

**Figure 6:**
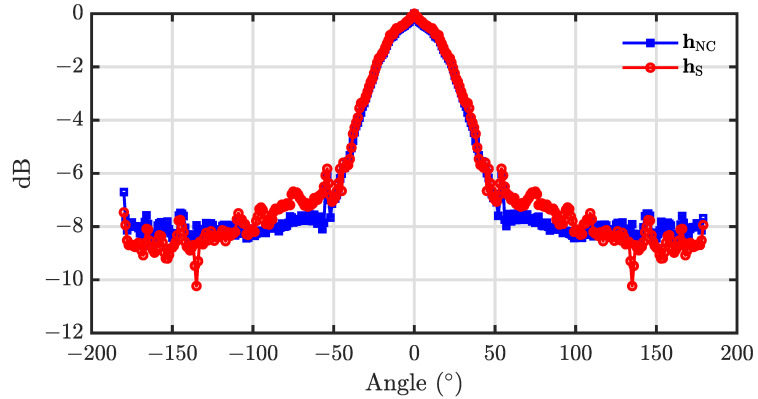
The normalized energy of the output signal of the beamformer $h_{\mathrm{NC}}\left(\omega \right)$ and $h_{S}\left(\omega \right)$ for the input signal from different direction. Conditions: N=3, M=7, R=20, r=0.02 m, and $\theta_{s}=0$.

For evaluating the performance of the beamformer $h_{\mathrm{NC}}\left(\omega \right)$, we simulated the room acoustic environments with the well-known image model [64,65]. The sampling rate is set to 8 kHz. The length, width and height of the simulated room are, respectively, 4 m, 4 m and 3 m. The position of the point in the simulated room is denoted as (x,y,z) with respect to a corner of the room which is considered the origin of the Cartesian coordinate system. A UCA with radius r=0.02 m and 7 microphones, whose center and the first microphone are at (2, 2, 1.5) (in meter, the same below) and (2.02, 2, 1.5), respectively, is considered in the simulation. The main lobe level to sidelobe level ratio, *R*, is set to 20 dB. 360 loudspeakers are simulated as sources of interest and placed at the same horizontal plane of the UCA with $1^{\circ }$ interval, and the distance between the loudspeakers and the center of the UCA is 1.5 m. The reflection coefficients of all six walls are set to 0.40 and the corresponding reverberation time $T_{60}\approx 110$ ms. The room impulse responses (RIRs) between every microphone and loudspeaker are generated using the image model. The output signal of each microphone is generated by convoluting a piece of the clean speech signal with the simulated RIRs. The normalized energy of the output signal of the beamformer $h_{\mathrm{NC}}\left(\omega \right)$ and $h_{S}\left(\omega \right)$ is presented in Figure 6. As seen from Figure 6, for input signal from different directions, the output energy of the beamformer $h_{\mathrm{NC}}\left(\omega \right)$ corresponding to the sidelobe region keeps flatter than that of the beamformer $h_{S}\left(\omega \right)$.

**Figure 7:**
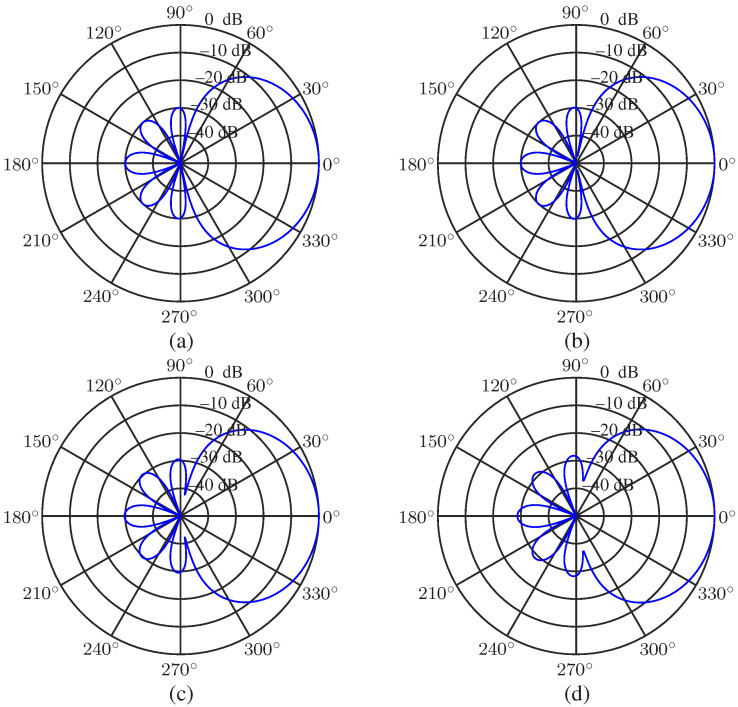
Beampatterns of the least square beamformer, $h_{\mathrm{LS}}\left(\omega \right)$, with different frequencies: (**a**) f=500 Hz, (**b**) f=1000 Hz, (**c**) f=2000 Hz, and (**d**) f=4000 Hz. Conditions: N=3, M=7, R=30, r=0.02 m, and $\theta_{s}=0$.

Now, we take a look at the beampatterns of the least square beamformer. Again, we use the setting of N=3, R=30 (dB), M=7, and r=0.02 m. The resulting beampatterns are shown in Figure 7, from which we can see the same phenomenon, i.e., the array beampattern is similar to the desired one at low frequencies, and it diverges from the desired one at high frequencies.

**Figure 8:**
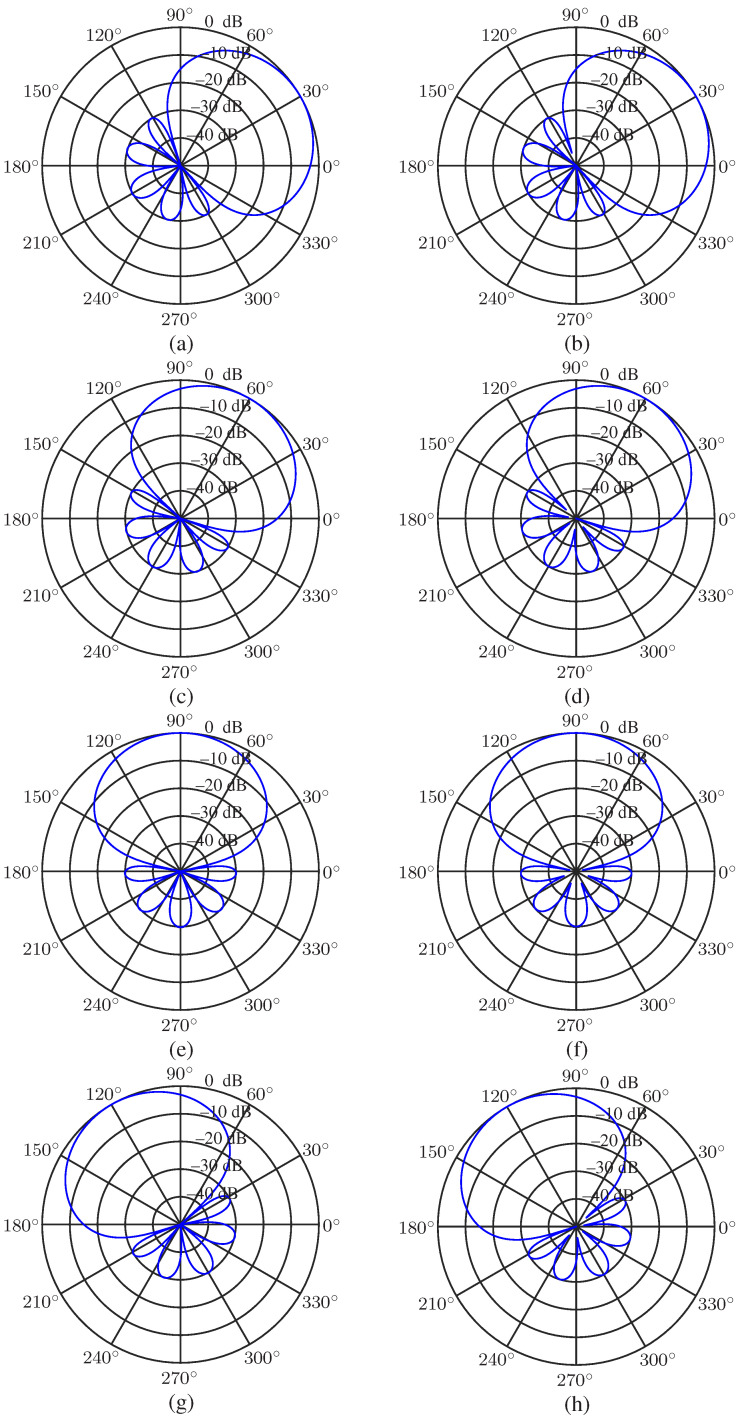
Beampatterns of the beamformers $h_{\mathrm{NC}}\left(\omega \right)$ and $h_{\mathrm{LS}}\left(\omega \right)$ with different values of $\theta_{s}$: (**a**) $h_{\mathrm{NC}}\left(\omega \right)$, $\theta_{s}=30^{\circ }$, (**b**) $h_{\mathrm{LS}}\left(\omega \right)$, $\theta_{s}=30^{\circ }$, (**c**) $h_{\mathrm{NC}}\left(\omega \right)$, $\theta_{s}=60^{\circ }$, (**d**) $h_{\mathrm{LS}}\left(\omega \right)$, $\theta_{s}=60^{\circ }$, (**e**) $h_{\mathrm{NC}}\left(\omega \right)$, $\theta_{s}=90^{\circ }$, (**f**) $h_{\mathrm{LS}}\left(\omega \right)$, $\theta_{s}=90^{\circ }$, (**g**) $h_{\mathrm{NC}}\left(\omega \right)$, $\theta_{s}=120^{\circ }$, and (**h**) $h_{\mathrm{LS}}\left(\omega \right)$, $\theta_{s}=120^{\circ }$. Conditions: N=3, M=7, R=30, r=0.02 m, and f=1000 Hz.

Using the same array and Chebyshev polynomial parameters as the last simulation, we study the steering ability of the null-constrained and least square beamformers. Figure 8 describes the beampatterns of the beamformers $h_{\mathrm{NC}}\left(\omega \right)$ and $h_{\mathrm{LS}}\left(\omega \right)$ with different direction of the desired signal, $\theta_{s}$ ($\theta_{s}=30^{\circ },60^{\circ },90^{\circ }$ and $120^{\circ }$), at frequency f=1000 Hz. As we can see, the beampattern of the proposed two beamformers can be steered to any direction without distortion.

**Figure 9:**
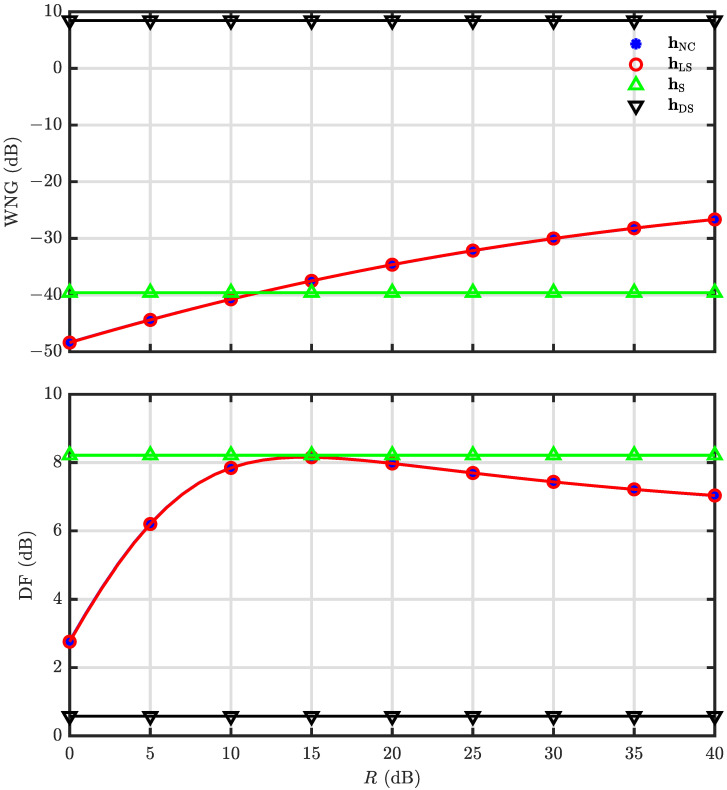
Performance of the beamformers $h_{\mathrm{NC}}\left(\omega \right)$ and $h_{\mathrm{LS}}\left(\omega \right)$ versus the parameter *R*: WNG (**top**), and DF (**bottom**). Conditions: N=3, M=7, f=1000 Hz, r=0.02 m, and $\theta_{s}=0$.

**Figure 10:**
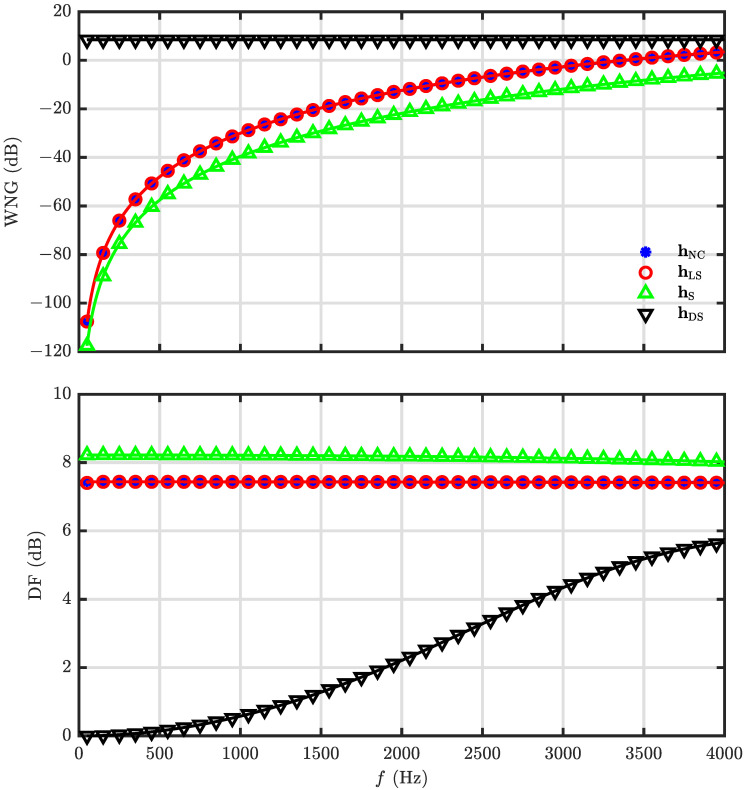
Performance of the beamformers $h_{\mathrm{NC}}\left(\omega \right)$ and $h_{\mathrm{LS}}\left(\omega \right)$ versus frequency: WNG (**top**), and DF (**bottom**). Conditions: N=3, M=7, R=30, r=0.02 m, and $\theta_{s}=0$.

The WNG and DF of the null constrained and least square beamformers are investigated in our simulations, and the parameter settings are the same as in the last simulation. Figure 9 shows the WNG and DF with respect to *R* at f=1000 Hz, and the results of the DS and superdirective beamformers are also included. As seen, the DF of the null constrained and least square beamformers increase with *R* first and then decreases, which is always less than that of the superdirective beamformer as expected. The WNG, however, increases with *R* monotonically. The WNG and DF of $h_{\mathrm{NC}}\left(\omega \right)$ and $h_{\mathrm{LS}}\left(\omega \right)$ as functions of frequency with N=3 and R=30 are plotted in Figure 10. It is observed that the WNG is very low at low-frequency bands, which is the motivation of the development of the minimum norm solution.

### 5.3. Performance of the Minimum Norm and Combined Solutions

**Figure 11:**
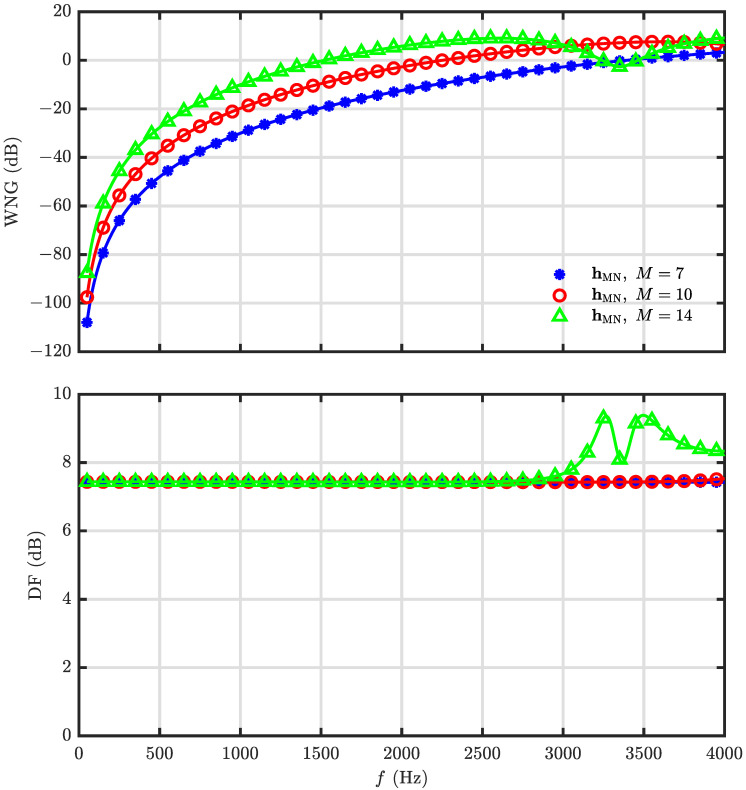
Performance of the beamformer $h_{\mathrm{MN}}\left(\omega \right)$ versus frequency with different microphone number, *M*: WNG (**top**), and DF (**bottom**). Conditions: N=3, R=30, δ=0.0174 m, and $\theta_{s}=0$.

**Figure 12:**
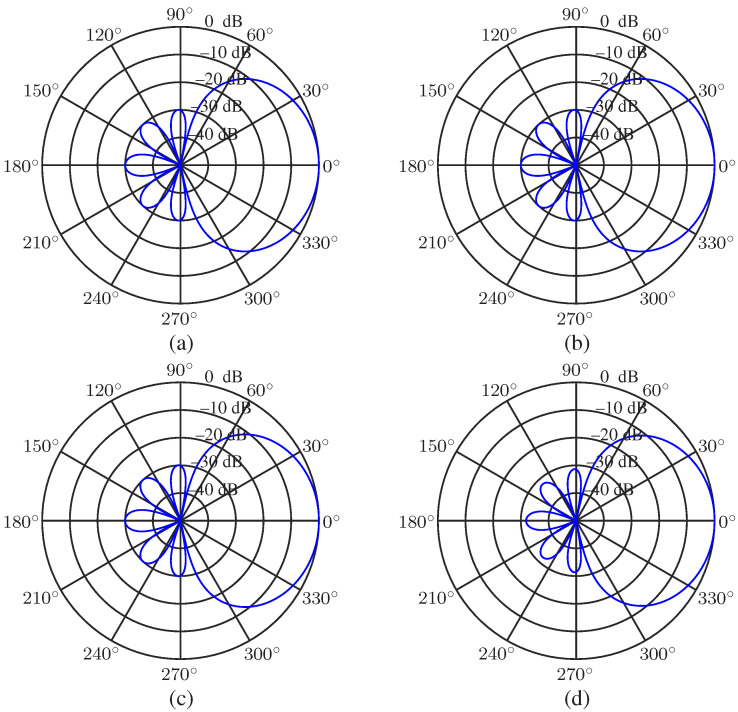
Beampatterns of the beamformer $h_{\mathrm{MN}}\left(\omega \right)$ with different frequencies: (**a**) f=500 Hz, (**b**) f=1000 Hz, (**c**) f=2000 Hz, and (**d**) f=4000 Hz. Conditions: N=3, M=10, R=30, δ=0.0174 m, and $\theta_{s}=0$.

This simulation is devoted to the investigation of the minimum norm solution of the DMA. The desired beampattern is with N=3 and R=30. The WNG and DF versus frequency for different microphone numbers, M=7 (i.e., the null constrained solution), 10, 14, are plotted in Figure 11. Note that the inter-element spacing (which is $\delta =2r\sin\left(\frac{\pi }{M}\right)$ for a UCA, and *r* denotes the radius. When r=0.02 m and M=7, δ=0.0174 m) keeps the same for different microphone number. From Figure 11, it is observed that the WNG increases with the microphone number, which exactly corresponds to the analysis. Figure 12 plots the beampatterns of the minimum norm beamformer with M=10 at different frequencies (f=500,1000,2000 and 4000 Hz). Comparing with the desired beampattern in Figure 2d, we observe that, though the WNG is improved at low-frequency bands, the beampattern deviates from the target at high-frequency bands also as the beamformers $h_{\mathrm{NC}}\left(\omega \right)$ and $h_{\mathrm{LS}}\left(\omega \right)$. For overcoming this drawback, the combined solution is derived.

**Figure 13:**
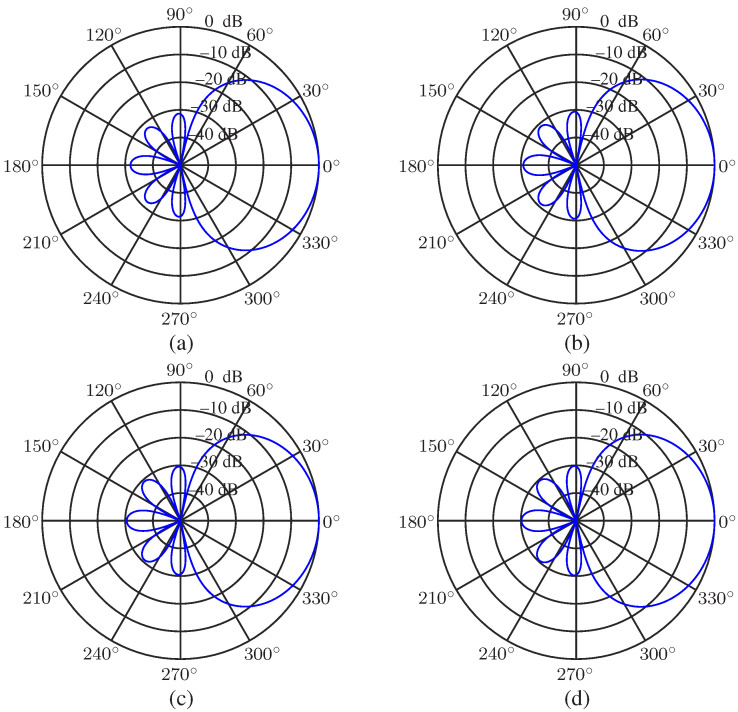
Beampatterns of the beamformer $h_{C}\left(\omega \right)$ with different values of μ: (**a**) μ=1.0, (**b**) μ=0.99, (**c**) μ=0.98, and (**d**) μ=0.95. Conditions: N=3, M=10, R=30, δ=0.0174 m, f=1000 Hz, and $\theta_{s}=0$.

**Figure 14:**
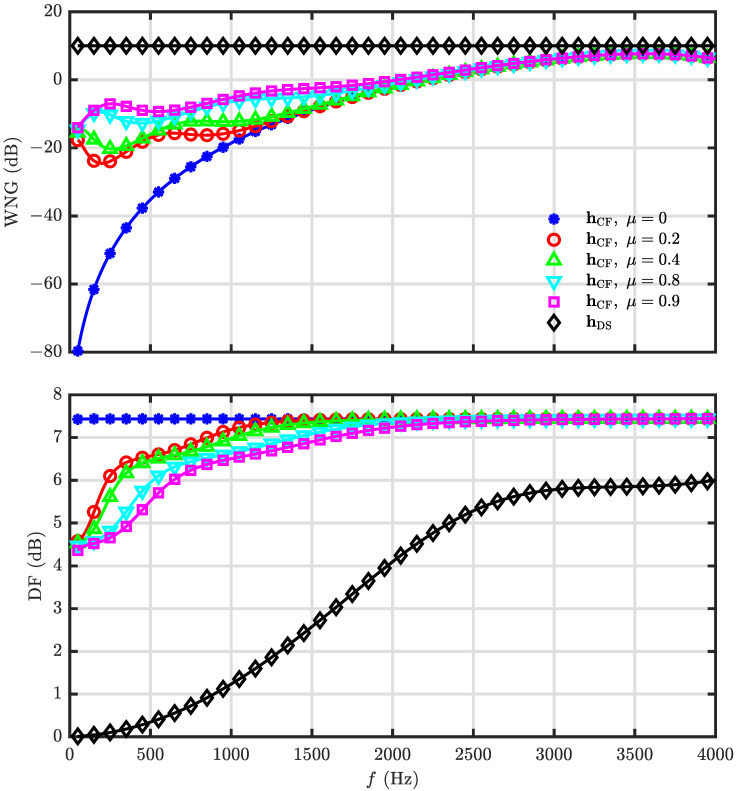
Performance of the beamformer $h_{\mathrm{CF}}\left(\omega \right)$ versus frequency with different values of the parameter μ: WNG (**top**), and DF (**bottom**). Conditions: N=3, M=10, R=30, δ=0.0174 m, and $\theta_{s}=0$.

**Figure 15:**
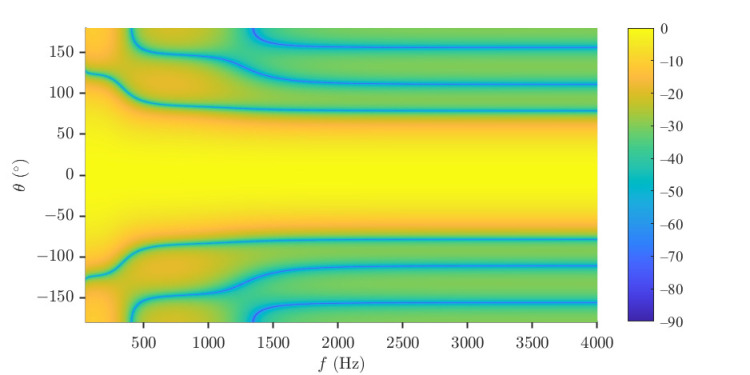
Beampattern of the beamformer $h_{\mathrm{CF}}\left(\omega \right)$ versus frequency. Conditions: N=3, M=10, R=30, μ=0.4, δ=0.0174 m, and $\theta_{s}=0$.

In this part, the combined solutions are studied. Again, the desired beampattern is with N=3 and R=30, and *M* is selected to be 10. At first, the beampatterns of $h_{C}\left(\omega \right)$ with different values of μ (μ=1,0.99,0.98,0.95) are investigated at f=4000 Hz, and the results are shown in Figure 13. As seen, as μ decreases, the approximation of the array beampattern to the desired one goes better. Then, the performance of the beamformer $h_{\mathrm{CF}}\left(\omega \right)$ with μ=0.2,0.4,0.8,0.9 is investigated. The WNG and DF versus frequency are plotted in Figure 14, including the results of the DS and least square (μ=0) beamformers. As expected, the WNG increases and the DF decreases with the increase of μ. Figure 15 plots the 3-D beampattern of the beamformer $h_{\mathrm{CF}}\left(\omega \right)$ versus the frequency with μ=0.4 and $\theta_{s}=0$. we can make compromises among the WNG, DF and beampattern distortion by adjusting the parameter μ flexibly. As seen from Figure 15, the level of different sidelobe keeps the same at relatively high frequencies, and we can make compromises among the WNG, DF and beampattern distortion by adjusting the parameter μ flexibly.

At last, we evaluate the performance of the beamformer $h_{\mathrm{CF}}\left(\omega \right)$ in the simulated room acoustic environment with image model. A UCA with radius r=0.039 m and 14 microphones, whose center and first microphone are, respectively, at (2, 2, 1.5) and (2.039, 2, 1.5), is considered in the simulation, and the loudspeaker is placed at (3.5, 2, 1.5). The reflection coefficients of all six walls are set to 0.85 and the corresponding reverberation time $T_{60}\approx 350$ ms. The simulated RIRs are divided into two parts, i.e., the direct path and reflections, based on which two metrics are defined, i.e., the direct-path-signal-to-noise ratio (DSNR), and the direct-path-signal-to-reverberation ratio (DSRR). The spatially and temporally white Gaussian noise is added to the microphone signals with DSNR=20 dB. The input DSRR for our simulation setup is approximately 2.98 dB. The result is presented in Table 2. It can be seen that the DSNR increases and DSRR decreases with the increase of μ, which is consistent with the analysis. One should note that, the output DSNRs of the beamformer $h_{\mathrm{CF}}\left(\omega \right)$ with μ=0.2,0.4,0.8,0.9 are smaller than the input DSNR because negative WNG of the beamformer $h_{\mathrm{CF}}\left(\omega \right)$ in relatively low frequency, which can be seen in Figure 14.

## 6. Conclusions

In this paper, by investigating the structure of the frequency-independent beampattern of a theoretical *N*th-order differential beamformer and exploiting the Chebyshev polynomials, the desired beampattern design methods which can exactly control the main lobe beamwidth and sidelobe level and obtain minimum main lobe beamwidth with a given sidelobe level are proposed. The designed beam pattern can achieve a narrower main lobe beamwidth than that of the hypercardioid beampattern with the same order, and the relationship among the main lobe beamwidth, sidelobe level and polynomial order is deduced. To obtain the designed desired beampattern, the null constrained beamformer, $h_{\mathrm{NC}}\left(\omega \right)$, and least square beamformer, $h_{\mathrm{LS}}\left(\omega \right)$, are developed, which can approximate the desired beampattern very well and consequently have a frequency-invariant spatial response at relatively low frequencies. However, the beampatterns of $h_{\mathrm{NC}}\left(\omega \right)$ and $h_{\mathrm{LS}}\left(\omega \right)$ deviate from the desired one at high frequencies. In addition, the WNG of $h_{\mathrm{NC}}\left(\omega \right)$ and $h_{\mathrm{LS}}\left(\omega \right)$ is very low at low frequencies, which means serious white noise amplification. So, the minimum norm solution $h_{\mathrm{MN}}\left(\omega \right)$ and combined solutions $h_{C}\left(\omega \right)$ and $h_{\mathrm{CF}}\left(\omega \right)$ are proposed, which can achieve a flexible compromise among the WNG, DF and beampattern distortion by adjusting the microphone number *M* and the parameter μ.

## Figures and Tables

**Table 1:** The Mapping Relationship Between θ and *x*.

θ	0	π
$x=c_{1}\cos\theta +c_{2}$	$x_{0}$	−1

**Table 2:** Performance of beamformer $h_{\mathrm{CF}}\left(\omega \right)$ with different value of μ. Conditions: $T_{60}\approx 350$ ms, M=14 and r=0.039 m, the input DSNR and DSRR are, respectively, 20 dB and 2.98 dB.

Beamformer	DSNR (dB)	DSRR (dB)
$h_{\mathrm{CF}}\left(\omega \right)$, μ=0.2	5.14	4.89
$h_{\mathrm{CF}}\left(\omega \right)$, μ=0.4	8.24	4.74
$h_{\mathrm{CF}}\left(\omega \right)$, μ=0.8	14.24	4.39
$h_{\mathrm{CF}}\left(\omega \right)$, μ=0.9	15.81	4.24

## Data Availability

The data presented in this study are available on request from the corresponding author.
